# Inverted internal limiting membrane flap technique versus complete internal limiting membrane peeling in large macular hole surgery: a comparative study

**DOI:** 10.1186/s12886-019-1294-8

**Published:** 2020-01-06

**Authors:** Prithvi Ramtohul, Eric Parrat, Danièle Denis, Umberto Lorenzi

**Affiliations:** 1 Centre Hospitalier Universitaire de l’Hôpital Nord, chemin des Bourrely, 13015 Marseille, France; 2 Centre Hospitalier Universitaire de Rouen, 37 Boulevard Gambetta, 76000 Rouen, France; 3 Department of Ophthalmology, University Medical Center of Pointe-à-Pitre, Les Abyme, Guadeloupe

**Keywords:** Internal limiting membrane, Inverted ILM flap technique, ILM peeling, Macular hole, Vitrectomy

## Abstract

**Background:**

To compare the anatomical and functional outcomes of the inverted internal limiting membrane (ILM) flap technique and the complete ILM removal in the treatment of large stage 4 macular hole (MH) > 400 μm and to evaluate reconstructive anatomical changes in foveal microstructure using spectral-domain optical coherence tomography.

**Methods:**

This is a retrospective, consecutive, nonrandomized comparative study of patients affected by idiopathic, myopic or traumatic stage 4 MH (minimum diameter > 400 μm) treated with 25-gauge pars-plana vitrectomy with either complete ILM peeling (*n* = 23, Group 1) or inverted ILM flap technique (*n* = 23, Group 2), between August 2016 and August 2018. Main outcomes measured were the MH closure rate assessed by spectral-domain optical coherence tomography and the best-corrected visual acuity (BCVA) at six months. Foveal microstructure reconstructive changes were evaluated using SD-OCT to determine predictive factors of postoperative BCVA.

**Results:**

Closure of MH was achieved in 16/23 cases of Group 1 (70%) and in 22/23 cases of the Group 2 (96%). Surgical failure was reported in 6 cases of Group 1 and 1 case of Group 2. The MH closure rate was significantly higher with the inverted ILM flap technique (*P*-value = 0.02). Average BCVA (LogMAR) changed from 1.04 ± 0.32 to 0.70 ± 0.31 in Group 1 and from 0.98 ± 0.22 to 0.45 ± 0.25 in Group 2 (*P*-value = 0.005) at 6 months. Improvement in BCVA (> 0.3 LogMAR units) was statistically higher in the Group 2 (*P*-value = 0.03). Restoration of foveal microstructure was significantly higher in the Group 2 at 6 months (52% vs 9%, *P*-value < 0.01). In Group 2, the integrity of the external limiting membrane at 3 months postoperatively was the only significant feature correlated with postoperative BCVA at 6 months (*r* = 0.562; *P*-Value = 0.01, forward stepwise regression analysis).

**Conclusion:**

Inverted ILM flap technique is more effective than the classic ILM peeling for the closure of large stage 4 MHs > 400 μm, improving both anatomical and functional outcomes. Early recovery of the external limiting membrane at 3 months is a positive predictive value of postoperative BCVA 6 months after inverted ILM flap technique.

## Background

A macular hole (MH) is a retinal defect that arises at the level of the internal limiting membrane (ILM) and extends up to, but not including, the retinal pigment epithelium (RPE), in the foveolar region. Primary MH, resulting from vitreo-macular tractions, either tangential and anteroposterior, typically occurs in the elderly, with a prevalence ranging from 0,2% to 0,8% in the general population [1]. MH represents a vision-threatening event requiring a prompt treatment. MH surgery was first described by Kelly and Wendel in 1991 [2]. Ever since, progression of surgical techniques, instrumentations and diagnostic tools have enhanced the anatomical and functional outcomes of MH surgery.

Pars-plana vitrectomy (PPV) combined with ILM removal and gas tamponade is considered as the standard procedure in the treatment of MH. Fundamental principles of the surgery efficacy are multifactorial and rest on the elimination of vitreo-macular tractional forces, the extensibility of the retina and the MH bridging by Müller cell gliosis activation [3].

Although the MH closure rate reaches approximately 90% with the standard procedure, some cases are more challenging and display a worsened prognosis [4]. In fact, large MHs’ diameters > 400 μm, chronic MHs, and secondary MHs, resulting from ocular trauma, severe myopia, retinal detachment or proliferative vitreoretinopathy, present with poor anatomical and functional outcomes [5]. In 2010, Michalewska et al. introduced the inverted ILM flap technique, improving both visual acuity and closure success of large idiopathic MHs and myopic MHs [6]. More recently, Rizzo et al. found out, in a large comparative study, a 95.6% and a 88.4% closure rate respectively in idiopathic MHs > 400 μm and myopic MHs with the inverted ILM flap procedure [7].

Several studies reported spectral-domain optical coherence tomography (SD-OCT) structural changes in the photoreceptor layers of eyes with surgically closed MH, including disruption of the ellipsoid zone (EZ) and external limiting membrane (ELM). However, only a few studies evaluated the reconstructive change in foveal anatomy in MHs surgically closed with the inverted ILM flap technique, suggesting that the restoration of the ELM is a critical structural feature significantly correlated with improved postoperative BCVA [8].

The purpose of this study was to compare the anatomical and functional outcomes of the inverted ILM flap technique and the complete ILM removal in the treatment of large stage 4 MHs (diameter > 400 μm) and to evaluate reconstructive anatomical changes in foveal microstructure using SD-OCT.

## Methods

This is a retrospective study based on the review of medical records of forty-six eyes of 46 consecutive patients affected by large stage 4 MH, who underwent a surgical treatment at Clinique Les Eaux Claires and University Medical Center of Pointe-à-Pitre in Guadeloupe between August 2016 and August 2018. The study complied with the Declaration of Helsinski. Ethical committee approval was not required due to the retrospective nature of this study. Oral informed consent for participation was obtained from all patients.

The inclusion criteria were as follows: minimum MH > 400 μm, age > 18 and at least 6 months of follow-up. Exclusion criteria were the presence of a chorioretinal atrophy involving the fovea (diagnosed on SD-OCT as a [1] region of hypertransmission of at least 250 μm in diameter, [2] a zone of attenuation or disruption of the RPE of at least 250 μm in diameter, [3] evidence of overlying photoreceptor degeneration, and [4] absence of scrolled RPE or other signs of an RPE tear), previous retinal surgery, presence of choroidal neovascularization, diabetic retinopathy or other ocular conditions that could influence the BCVA, except lens opacity.

At baseline and at every follow-up visit (1 month, 3 months and 6 months after surgery), all patients underwent a complete ophthalmologic examination, including measurement of the BCVA, slit-lamp examination and biomicroscopy of the posterior segment using a slit-lamp with a non-contact 90D lens. BCVA measured with a Snellen chart was converted to logarithm of minimum angle of resolution (LogMAR) for statistical analysis. MH diameter and outer retinal layers were evaluated at the same time by SD-OCT (Heidelberg Engineering GmbH, Heidelberg, Germany). Tracking system was used for the follow-up, allowing accurate and repeatable measurements.

Microstructural imaging analysis of the fovea was performed using SD-OCT. Restoration of the photoreceptor layer was evaluated as recovery of the continuous back-reflection lines corresponding to the EZ and the ELM. We defined the continuous restoration of the EZ and the ELM as EZ (+) and ELM (+) respectively. The postoperative photoreceptor recovery, corresponding to the EZ and the ELM lines, was evaluated at every follow-up visit (1 month, 3 months, 6 months). Two masked investigators (R.R. and T.A.) evaluated the SD-OCT images. In case of disagreement, a third investigator (M.S.) was referred for final decision.

PPV with conventional ILM peeling combined with C2F6 gas (perfluoroethane) tamponade was conducted in 23 eyes (Group 1: ILM peeling group) between August 2016 and August 2017, while PPV with inverted ILM flap technique and C2F6 gas tamponade was performed in 23 eyes (Group 2: ILM flap group) between August 2017 and August 2018. Cataract surgery was simultaneously performed in 13 eyes (7 eyes in the Group 1 and 6 eyes in the Group 2).

All patients had been operated by one of two experienced vitreoretinal surgeons (U.L. and E.P.).

### Surgical techniques

#### Complete ILM removal

A complete pars-plana vitrectomy was performed using the 25-gauge Stellaris PC System (Bausch and Lomb, Rochester, NY, USA), sutureless, and the Ocular Landers Wide Field Vitrectomy Lens (Ocular Instruments, Bellevue, Washington, USA). After this, the ILM was stained with ILM Blue injection (DORC, Aqumen Biopharmaceutical KK, Fukuoka, Japan) above the macula, followed by lavage. The ILM was peeled off centripetally, approximately 2 disk diameters around the MH. At the end of the surgery, the eye was filled with perfluoroethane (C2F6, 16%). The patients were instructed to maintain a face-down position for 5 days.

#### ILM flap technique

It was performed according to the original report of Michalewska *et al* [6]. During ILM peeling around the MH, the ILM flap was not entirely removed from the retina but a remnant was left attached to the edges of the MH. The ILM flap was approximately 1 or 1.5 diameter of the MH. It was lifted, inverted and pushed into the MH gently with forceps (25-gauge Eckardt End-gripping Forceps, Dutch Ophtalmic, USA) to fill the entire MH. Fluid-air exchange was performed using a low intraocular pressure and passive aspiration to avoid turbulence that could cause a flap displacement. At the end of the surgery, the eye was filled with perfluoroethane (C2F6, 16%). The patients were instructed to maintain a face-down position for 5 days.

### Statistical analysis

Statistical analysis was performed using JMP statistical analysis software, version 5.01 J (SAS Institute, Cary, NC) to analyse the differences in baseline characteristics, anatomical outcomes including macular hole closure and functional outcomes of BCVA between ILM peeling group (Group 1) and ILM flap group (Group 2). The BCVA was recorded as decimal value and converted to LogMAR for statistical analysis.

The Wilcoxon rank test was used to compare pre- and postoperative BCVA. The Chi2-test was used to compare MH closure rates after surgery and improvement in BCVA (> 0,3 LogMAR units) between 2 groups. The comparison of BCVA in the two groups was analysed in the Mann-Whitney U-test. Kruskal-Wallis 1-way analysis of variance (ANOVA) was performed followed by pairwise multiple comparison using Dunn’s method to assess the association between visual outcomes and the degree of recovery of the photoreceptor layer. The sample size was determined assuming that the inverted ILM flap technique was more effective for achieving large MH closure than classic ILM peeling (95% vs 85%) based on literature data. A sample size of 60 eyes was required to power the study comparison to 80% (with a 95% confidence interval). A *P*-value < 0.05 was considered to be statistically significant.

## Results

Twenty-three eyes of 23 consecutive patients operated with ILM removal (Group 1) and twenty-three eyes of 23 consecutive patients treated with inverted ILM flap technique (Group 2) were included in this study. Patients’ characteristics are shown in Table 1.

**Table 1:** Patient characteristics. Group 1: ILM Removal Group and Group 2: Inverted ILM flap Group

Patient no.	Age range	Eye	Preoperative lens status	MH diameter (μm)	Operative procedure	Tamponade	Preoperative BCVA (decimal)
Group 1							
1	70–74	R	IOL	598	PPV, PL	C2F6	0.2
*2*	75–79	R	IOL	652	PPV, PL	C2F6	0.01
3	75–79	L	IOL	479	PPV, PL	C2F6	0.2
4	60–64	R	Phake	753	PPV, PL, PEA, IOL	C2F6	0.1
5	75–79	R	IOL	451	PPV, PL	C2F6	0.1
6	65–69	L	IOL	505	PPV, PL	C2F6	0.2
7	55–59	L	IOL	697	PPV, PL	C2F6	0.05
8	60–64	R	IOL	500	PPV, PL	C2F6	0.2
9	65–69	L	Phake	1028	PPV, PL, PEA, IOL	C2F6	0.05
10	70–74	R	Phake	479	PPV, PL, PEA, IOL	C2F6	0.15
11	65–69	L	Phake	401	PPV, PL, PEA, IOL	C2F6	0.2
12	70–74	R	IOL	603	PPV, PL	C2F6	0.1
13	60–64	R	IOL	401	PPV, PL	C2F6	0.05
14	70–74	L	IOL	654	PPV, PL	C2F6	0.2
15	75–79	L	IOL	400	PPV, PL	C2F6	0.1
16	50–54	R	Phake	879	PPV, PL, PEA, IOL	C2F6	0.05
17	60–64	R	IOL	695	PPV, PL	C2F6	0.05
18	65–69	R	IOL	450	PPV, PL	C2F6	0.05
19	60–64	L	IOL	508	PPV, PL	C2F6	0.1
20	70–74	R	IOL	400	PPV, PL	C2F6	0.15
21	65–69	R	Phake	400	PPV, PL, PEA, IOL	C2F6	0.1
22	65–69	R	IOL	650	PPV, PL	C2F6	0.05
23	60–64	R	Phake	621	PPV, PL, PEA, IOL	C2F6	0.1
Group 2							
1	65–69	L	IOL	426	PPV, ILM	C2F6	0.12
2	70–74	L	IOL	432	PPV, ILM	C2F6	0.3
3	65–69	R	Phake	502	PPV, ILM, PEA, IOL	C2F6	0.16
4	65–69	R	IOL	571	PPV, ILM	C2F6	0.1
5	65–69	R	IOL	575	PPV, ILM	C2F6	0.08
6	70–74	L	IOL	573	PPV, ILM	C2F6	0.1
7	75–79	R	Phake	593	PPV, ILM, PEA, IOL	C2F6	0.16
8	65–69	L	IOL	640	PPV, ILM	C2F6	0.04
9	70–74	R	IOL	642	PPV, ILM	C2F6	0.16
10	60–64	R	IOL	694	PPV, ILM	C2F6	0.08
11	65–69	L	IOL	699	PPV, ILM	C2F6	0.08
12	60–64	L	Phake	691	PPV, ILM, PEA, IOL	C2F6	0.04
13	65–69	R	Phake	739	PPV, ILM, PEA, IOL	C2F6	0.08
14	45–49	L	IOL	746	PPV, ILM	C2F6	0.16
15	75–79	L	Phake	798	PPV, ILM, PEA, IOL	C2F6	0.1
16	60–64	R	IOL	792	PPV, ILM	C2F6	0.16
17	75–79	L	IOL	833	PPV, ILM	C2F6	0.16
18	70–74	R	IOL	891	PPV, ILM	C2F6	0.1
19	40–44	L	IOL	1159	PPV, ILM	C2F6	0.1
20	55–59	R	Phake	674	PPV, ILM, PEA, IOL	C2F6	0.05
21	75–79	R	IOL	721	PPV, ILM	C2F6	0.1
22	55–59	R	IOL	438	PPV, ILM	C2F6	0.25
23	65–69	R	IOL	524	PPV, ILM	C2F6	0.1

Abbreviations: *BCVA* best-corrected visual acuity, *C2F8* hexafluoroethane, *ILM* Inverted ILM flap, *IOL* intraocular lens, *LogMAR* logarithm of minimum angle of resolution, *MH* macular hole, *PEA* phacoemulsification, *PPV* pars plana vitrectomy

The comparison of preoperative data between the two groups are shown in Table 2. There were no statistically significant differences in age ranges (*P*-value = 0.14), sex (*P*-value = 0.37), duration of symptoms (*P*-value = 0.54), type of MH (idiopathic: P-value = 0.75/myopic: *P*-value = 0.44/traumatic: *P*-value = 0.64) or preoperative BCVA (*P*-value = 0.48). The mean minimal MH diameter was higher in the inverted ILM flap group, but the difference was not statistically significant (Peeling group: 574.09 ± 164.68 μm; ILM flap group: 657.33 ± 172.36 μm, *P*-value = 0.09).

**Table 2:** Comparison of preoperative study participant data

Characteristics	Group 1 (ILM Removal)	Group 2 (Inverted ILM Flap)	*P*-value
No. of eyes	23	23	
Age (years; mean ± SD)	65.69 ± 10.25	68.03 ± 9.50	0.14
Gender (male/female)	16 (69%)/ 7 (31%)	13 (56%) / 10 (44%)	0.37
Duration of symptoms (weeks; mean ± SD)	13.2 ± 3.6	12.5 ± 4.1	0.54
Preoperative lens status, *n* (%)			
Phakic	7 (30%)	6 (26%)	0.75
Pseudophakic	16 (70%)	17 (74%)	0.75
Preoperative BCVA (LogMAR; mean ± SD)	1.04 ± 0.32	0.98 ± 0.22	0.48
MH diameter (μm; mean ± SD)	574.09 ± 164.68	657.33 ± 172.36	0.09
Type			
Idiopathic	17	16	0.75
Myopic	3	5	0.44
Traumatic	3	2	0.64

Abbreviations: *BCVA* best-corrected visual acuity, *LogMAR* logarithm of minimum angle of resolution, *MH* macular hole

Surgery was considered successful when a complete closure of the MH was achieved (defined as the absence of a neurosensory defect over the retina). Flat-open and elevated-open MH were considered surgical failures. At six months of follow-up, SD-OCT showed MH closure in 16 cases in Group 1 (70%) and 21 cases in Group 2 (91%) after one single surgery. No cases of flat-open or elevated-open MH were found at 6 months.

Seven surgical failures occurred in the Group 1. The mean diameter of the MH was 643.83 ± 157.37 μm. An additional surgery was performed for three patients but the MH remained open at 6 months. In Group 2, there were 2 surgical failures (Mean diameter = 573 μm ± 2 μm). One of these patients was reoperated and presented a MH closure after a 6-month follow-up.

Phacoemulsification was performed in combination with PPV in 7 cases (30%) from the Group 1 and 6 cases (26%) from the Group 2. Therefore, each patient included was pseudophakic at the end of the follow-up.

Postoperative data are shown in Table 3. No statistically significant differences were found in the postoperative BCVA at 1 and 3 months (*P*-value > 0.05 respectively). Mean BCVA at 6 months was 0.70 ± 0.31 LogMAR in the ILM peeling group and 0.45 ± 0.25 LogMAR in the ILM flap group (*P*-value < 0.01). The difference between preoperative and postoperative BCVA was statically significant within the Group 1 and the Group 2 (*P*-value < 0.001). The improvement in BCVA (> 0.3 LogMAR units) at 6 months was significantly higher in the ILM flap group (*P*-value = 0.03).

**Table 3:** Comparison of postoperative study participant data

Characteristics	Group 1 (ILM Removal)	Group 2 (Inverted ILM Flap)	*P*-value
Postoperative BCVA (LogMAR; mean ± SD)			
1 month	0.77 ± 0.31	0.74 ± 0.25	0.73
3 months	0.67 ± 0.33	0.67 ± 0.26	0.95
6 months	0.70 ± 0.31	0.45 ± 0.25	0.005
Improvement in BCVA	−0.35 ± 0.33	−0.54 ± 0.25	0.03
Primary MH closure rate	70% (16/23)	91% (21/23)	0.67
`Additional surgery	3 (No surgical success)	1 (One surgical success)	0.30
Final MH closure rate	70% (16/23)	96% (22/23)	0.02

Abbreviations *BCVA* best-corrected visual acuity, *LogMAR* logarithm of minimum angle of resolution, *MH* macular hole

The primary MH closure rate was 70% (16/23) in Group 1 and 91% (21/23) in Group 2 (*P*-value = 0.67). The final MH closure rates at 6 months remained unchanged at 70% (16/23) in Group 1. However, it reached 96% (22/23) in Group 2 and this difference was statistically significant (P-value = 0.02).

**Fig. 1:**
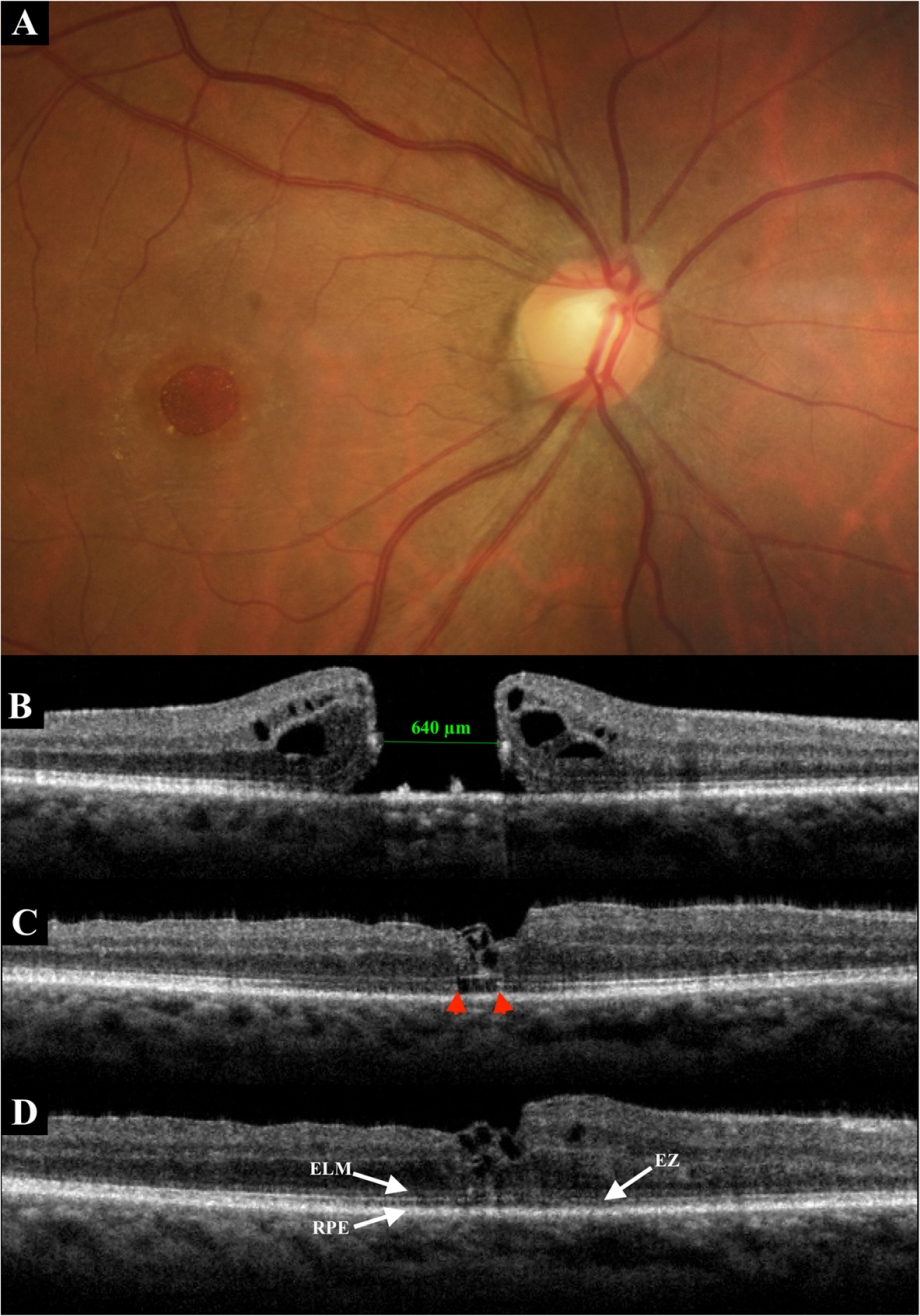
A 61-year-old man presented with idiopathic large MH. **a**. Fundus photography before surgery. **b**. Baseline SD-OCT. The minimum diameter was 640 μm. Preoperative BCVA was 0.04. **c**. SD-OCT at 1 month postoperatively. Inverted ILM flap technique was performed, with a W-shape closure (irregular closure) of the MH. SD-OCT showed the foveal hyperreflective tissue corresponding to a coiling of the flap. Disruption of the EZ (*between red arrowheads*) remained. The ELM line was almost complete. BCVA was 0.16. **d**. Six months after surgery, SD-OCT showed a persistent W-shape closure of the MH with a wrapped flap. There was a complete restoration of the ELM and EZ lines. The BCVA increased to 0.5

Postoperative recovery of the photoreceptor layers is shown in Table 4. SD-OCT examination in the Group 1 revealed ELM (+) in 0 (0%), 2 (9%), 4 (17%) patients at 1, 3 and 6 months respectively, and EZ (+) in 0 (0%), 1 (4%), 2 (9%) patients at 1, 3 and 6 months respectively. In Group 2, the ELM (+) was seen in 2 (9%), 12 (52%), 15 (65%) patients at 1, 3 and 6 months respectively and the EZ (+) was found in 1 (4%), 5 (21%), 12 (52%) patients at 1, 3 and 6 months respectively. Complete EZ restoration was not observed without complete restoration of the ELM. In both groups, restoration of the ELM preceded restoration of the EZ. Restoration of both ELM and EZ was significantly higher in the Group 2 (52% vs 9%, *P*-value < 0.01). Figure 1 highlights a case of complete ELM and EZ recovery following inverted ILM flap technique.

**Table 4:** Photoreceptor restoration during the follow-up period

	ELM (+)			EZ (+)		
	1 Month, n (%)	3 Months, n (%)	6 Months, n (%)	1 Month, n (%)	3 Months, n (%)	6 Months, n (%)
Group 1 (ILM Removal)	0 (0%)	2 (9%)	4 (17%)	0 (0%)	1 (4%)	2 (9%)
Group 2 (Inverted ILM Flap)	2 (9%)	12 (52%)	15 (65%)	1 (4%)	5 (21%)	12 (52%)

Abbreviations: *ELM (+)* Restoration of the external limiting membrane, *EZ (+)* Restoration of the ellipsoid zone, *ILM* Internal limiting membrane

In Group 2, the integrity of the ELM at 3 months postoperatively was the only significant feature correlated with postoperative BCVA at 6 months (*r* = 0.562; *P*-Value = 0.01, forward stepwise regression analysis).

## Discussion

Vitrectomy for MH, first described by Kelly and Wendel in 1991, has been the golden standard [2]. However, its effectiveness may be impaired in case of large, chronic or myopic MHs. Michalewska et al. proposed a novel surgical management, called inverted ILM flap technique, and demonstrated its efficacy in large idiopathic MHs (diameter > 400 μm) and in myopic MHs, improving both anatomical and functional outcomes [6].

In the current study, we included patients affected by idiopathic, myopic or traumatic stage 4 MHs. Our results showed that PPV associated with inverted ILM flap technique and gas tamponade displays a statistically higher closure rate (96% in the inverted ILM Group versus 70% in the ILM peeling Group, *P*-value < 0.05). Visual outcomes were also relevant in our study, as final BCVA was significantly higher with the inverted ILM flap technique (*P*-value < 0.01).

The exact mechanism of improved surgical results using the inverted ILM flap technique is not precisely understood. One theory is that the ILM flap may act as a scaffold for glial cells proliferation, resulting in MH filling with proliferating cells, which enhances MH closure. Another explanation might be that the ILM serves as a barrier, disabling the entrance of fluid from the vitreous cavity to the MH [9]. Moreover, the ILM flap is a source of Müller cells, which located at the surface of the ILM flap. Müller cells are important for assuming the role of optical fibers to transfer light from retinal surface to the photoreceptor cell layer. These findings explain why inducing Müller cells proliferation improves not only the MH closure rate but also the postoperative visual acuity [10].

Our findings are consistent with recent literature: Michalewska et al. [6], Kuriyama et al. [11], Mete et al. [12], and Ota et al. [13], found similar anatomical and functional outcomes for myopic MHs. In a recent comparative study conducted in a large series of patients, Rizzo et al. demonstrated the effectiveness of the inverted ILM flap technique compared with the ILM peeling for idiopathic and myopic MHs, improving both anatomical and visual outcomes. Among the patients affected by full-thickness MHs (diameter > 400 μm), the closure rate was 95.6% in the inverted ILM flap group and the mean postoperative BCVA was 0.43 LogMAR (*P*-value < 0.01) [7]. Our results are consistent with this study.

In our study, reconstruction of the foveal ELM 3 months postoperatively helps predict subsequent restoration of the ellipsoid zone and the potential for better visual outcomes. Restoration of both ELM and EZ was significantly higher with the inverted ILM flap technique. Integrity of the ELM at 3 months postoperatively was correlated with postoperative BCVA at 6 months (*r* = 0.562; *P*-Value = 0.01, forward stepwise regression analysis). Our results are consistent with Wakabayashi et al. suggesting that the ELM is a critical structural feature significantly correlated with postoperative BCVA [14].

Alternative surgical techniques have been proposed, trying to improve the original procedure described by Michalewska et al. Casini et al. compared the classic ILM flap technique with a modified and shorter ILM flap technique: the ILM flap remains attached to the retina only to the edges of the MH, looking like a funnel with the narrow opening attached to the retina, and no extra manipulations of the flap were performed. Their results showed no significantly difference in anatomical and functional outcomes between these two techniques [15]. Rossi et al. reported two surgical variations of the original ILM flap technique. In the “Cover technique”, the ILM was peeled centripetally and placed onto the MH in order to bridge the entire retinal defect with a single layer. In the “Fill technique”, the ILM was folded in multiple layers and packed within the MH using forceps. These two techniques showed similar closure rates and postoperative vision at 3 months. According to the authors, the Fill technique could be more efficient in closing larger MH [16]. Michalewska et al. reported a comparative study to determine if a temporal inverted ILM flap is as effective as the classic ILM flap technique for the repair of large stage IV idiopathic MH. They described a modified form where ILM peeling is restricted to the temporal side of the fovea only; the MH is then covered with the temporal ILM flap. Their results showed no significant differences in initial and final visual acuities between both techniques. The MH closure rate was 100% in both groups. They also noticed that the temporal inverted flap technique is associated with a lower incidence of dissociated optic nerve fiber layer (DONFL) by minimizing unnecessary surgical trauma to the nerve fiber layer [17]. Although DONFL has not been definitely associated with a decrease of visual acuity or microperimetry changes [18], there may be unknown effects on function that are not yet known. Temporal inverted ILM flap technique achieves satisfactory anatomical and functional results, as well as reduction of DONFL appearance, especially in the area of papillomacular bundle.

Our study has several limitations, including its retrospective nature, the absence of randomization for the type of treatment chosen, the relatively small number of patients and the follow-up period of 6 months, which was relatively short to evaluate the final BCVA. The power of our study could have been insufficient, considering the number of included patients, however, it was enough to draw a definitive conclusion with statistically significant results. This might suggest the clear superiority of the inverted ILM flap technique in the treatment of large MHs.

## Conclusions

In summary, inverted ILM flap technique is more effective than the classic ILM peeling for the closure of large stage 4 MHs > 400 μm, improving both anatomical and functional outcomes. We assessed foveal microstructural reconstructive changes following macular hole surgery. Restoration of both ELM and EZ was significantly higher with the inverted ILM flap technique. Early postoperative recovery of the ELM acts as a positive predictive value of final postoperative BCVA after inverted ILM flap technique.

## Data Availability

applicable. All data generated or analysed during the current study are included in this published article.
